# d-Limonene inhibits the occurrence and progression of LUAD through suppressing lipid droplet accumulation induced by PM_2.5_ exposure in vivo and in vitro

**DOI:** 10.1186/s12931-022-02270-9

**Published:** 2022-12-10

**Authors:** Tengteng Zhu, Yangyang Li, Tienan Feng, Yuqing Yang, Kai Zhang, Jing Gao, Xiaowei Quan, Ying Qian, Herbert Yu, Biyun Qian

**Affiliations:** 1 School of Public Health, Shanghai Tongren Hospital, Hongqiao International Institute of Medicine, Shanghai Jiao Tong University School of Medicine, Shanghai, 200052 China; 2 Shanghai Huangpu District Center for Disease Control and Prevention, Shanghai, 200002 China; 3 Cancer Epidemiology Program, University of Hawaii Cancer Center, Honolulu, HI 999203 USA; 4 Clinical Research Promotion and Development Center, Shanghai Hospital Development Center, 200040 Shanghai, China

**Keywords:** Lung adenocarcinoma, Lipid droplet, De novo lipogenesis, d-Limonene, PM_2.5_, Cancer prevention

## Abstract

**Background:**

PM_2.5_ exposure is associated with lung adenocarcinoma (LUAD), but the mechanism is unclear. The lack of understanding impedes our effort on prevention. This study examined a possible mechanism of lung cancer caused by PM_2.5_ exposure, and aimed to find a potential intervention for people living in PM_2.5_ polluted regions.

**Methods:**

Electron microscopy and oil-red staining were conducted to examine the lipid droplet accumulation. Masson’s trichrome staining, colony forming, scratch assay and transwell experiment were conducted to evaluate the effect of PM_2.5_ exposure and d-limonene intervention on the occurrence and progression of LUAD. Potential intervention targets were found by RNA-Seq and verified by luciferase reporter assay. MiR*-*195 KO mice constructed with CRISPR/Cas9 technology were used to investigate the pivotal role of d-limonene-miR-195-SREBP1/FASN axis. Cohort analysis of lung cancer patients, human LUAD tissues staining and human intervention trial were also conducted to validate the results of cell and animal experiments.

**Results:**

Our results showed that PM_2.5_ exposure induced accumulation of lipid droplets in LUAD cells which accompanied by increased malignant cellular behaviors. PM_2.5_ exposure led to cleaved N-SREBP1 translocation into nucleus, which activated the de novo lipogenesis pathway. Same changes were also observed in normal lung epithelial cells and normal lung tissue, and mice developed pulmonary fibrosis after long-term exposure to PM_2.5_. Furthermore, in a cohort of 11,712 lung cancer patients, significant lipid metabolism disorders were observed in higher PM_2.5_ polluted areas. In view of that, d-limonene was found to inhibit the changes in lipid metabolism through upregulating the expression of miR-195, which inhibited the expression of lipogenic genes (*SREBF1/FASN/ACACA*) specifically. And a small human intervention trial showed that serum miR-195 was upregulated after oral intake of d-limonene.

**Conclusion:**

Our findings reveal a new mechanism of pulmonary fibrosis and LUAD that is related to PM_2.5_ exposure-induced lipid droplet accumulation. We also demonstrate that d-limonene-miR-195-SREBP1/FASN axis is a potential preventive intervention for mediating the progression and development of LUAD induced by PM_2.5_ exposure.

*Trial registration* Chinese Clinical Trial Registry, ChiCTR2000030200. Registered 25 February 2020, http://www.chictr.org.cn/showproj.aspx?proj=48013

**Graphical Abstract:**

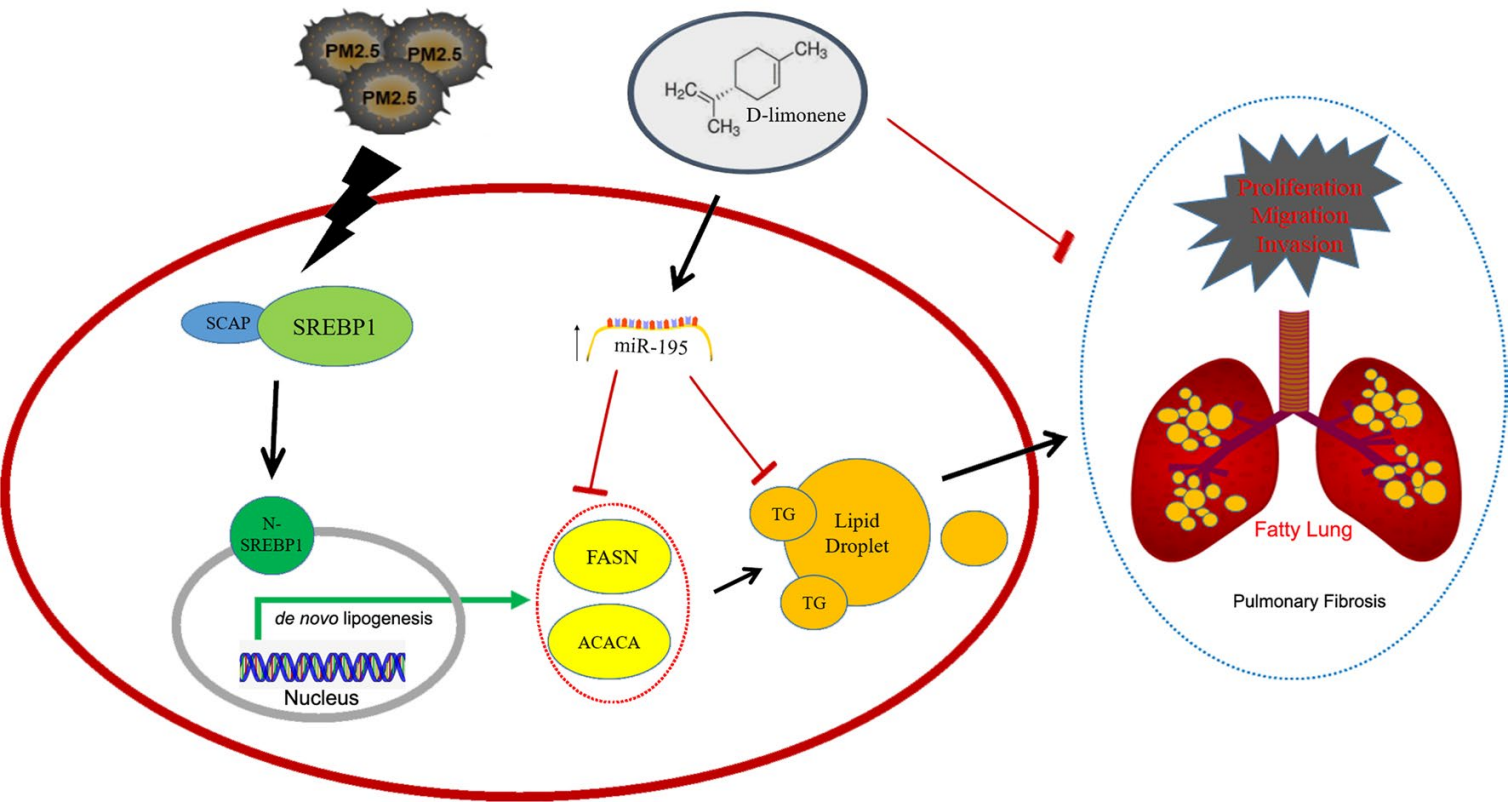

## Introduction

Lung cancer is the second most common malignant tumor after breast cancer and the leading cause of cancer-related death worldwide [1]. Due to the reduction in tobacco smoking, the incidence of lung squamous cell carcinoma (LUSC) had decreased, while the incidence of lung adenocarcinoma (LUAD) has increased in never-smokers [2]. Aside from smoking, epidemiological data show that outdoor ambient air pollution, especially fine particulate matter (PM_2.5_) pollution, is another risk factor for lung cancer and has been classified as a level I carcinogen by the International Agency for Research on Cancer [3]. Because of the small particle size (aerodynamic equivalent diameter ≤ 2.5 μm) and large surface area in a given concentration, PM_2.5_ can easily invade the human alveolar and blood circulation and carry a variety of hazardous materials, such as carcinogens, promoting the occurrence of disease. A meta-analysis indicated that lung cancer risk increased 9% when the PM_2.5_ concentration increased by 10 µg/m^3^ [4]. Based on a prospective study in the UK biobank, long-term exposure to PM_2.5_ was associated with lower lung function and increased chronic obstructive pulmonary disease (COPD) prevalence, and may increase the risk of lung cancer, especially in those with high genetic risk [5, 6]. Study on a Chinses cohort of 118,551 participants also provide strong evidence that high PM_2.5_ exposure leads to an elevated risk of lung cancer incidence and mortality [7]. However, the potential mechanism of PM_2.5_ induced pulmonary injury still needs further understanding.

Oxidative stress, inflammation response, autophagy and mitochondria damage have been the focus of PM_2.5_ induced lung injury and cardiovascular disease [8–11]. Recently, integrated metabolomics and proteomics analysis of human bronchial epithelial cells exposed to PM_2.5_ reveal significant alterations of many metabolic processes, such as amino acid metabolism, citric acid cycle and lipid metabolism [12]. Lipid metabolism alternation is another prominent character of cancer in additional to carbohydrate metabolism, and enhanced synthesis or uptake of lipids contributes to rapid cancer cell growth and tumor formation [13, 14]. Most studies carried on the effects of PM_2.5_ exposure on lipid metabolism have focused on cardiovascular and liver related diseases, especially in atherosclerosis, non-alcoholic steatohepatitis (NASH), hepatic fibrosis and hepatocellular carcinoma [15–18]. Nevertheless, studies about pulmonary injury caused by PM_2.5_ exposure induced-lipid metabolism disorder are scarce.

In the present study, we integrated LUAD/human bronchial cells, wide type/knock out mice models and population intervention trial to comprehensively study the potential biological mechanism of PM_2.5_ exposure on the occurrence and progression of LUAD through the lipid metabolism disorder pathway. We identified de novo synthesis of lipids pathway that play critical roles in mediating pulmonary injury induced by PM_2.5_ exposure. And increased lipid droplet formation was observed in cancer and normal cells respectively, which we called them ‘Fatty Lung’. We also found that D-limonene could inhibit the occurrence and progression of LUAD through inhibiting the activity of lipogenic genes. Collectively, our study reveals a possible mechanism of lung injury caused by PM_2.5_ exposure and suggests a novel low-cost preventive intervention for people living in PM_2.5_ polluted regions.

## Materials and methods

### PM_2.5_ sample collection and preparation

The sampling site was located at Shanghai Jiao Tong University School of Medicine in Shanghai City, China. PM_2.5_ was sampled continuously using an Echo PM Low Volume Sampler near the air intake for the exposure chambers (MSP HFI-129, China). Every 3 days, a new filter was used for sampling. Additionally, field blanks were used to control for possible contamination during the sampling procedures. After sampling, the filters were cut into tiny pieces and placed in an ultrasonicator for 2 h. After filtration, PM_2.5_ solution was purified in a vacuum freeze drier for 7 days, and PM_2.5_ powder was stored in an ultracold storage freezer. Inductively Coupled Plasma Mass Spectrometry (ICP-MS), Gas Chromatography Mass Spectrometer (GC–MS) and Carbon analyser were used to analysis the compositions of PM_2.5_ powder.

### Cell culture and treatments

Human lung adenocarcinoma cell lines (A549, NCL-H1975) and immortalized human bronchial epithelial cell line (Beas-2b) were obtained from the American Type Culture Collection. Cells were kept in DMEM/high-glucose medium supplemented with 10% FBS and 1% penicillin/streptomycin at 37 °C in a 5% CO_2_ humidified culture incubator. According to our previous studies [19, 20], cells were exposed to PM_2.5_ or d-limonene for 48 h at a final concentration of 100 µg/mL or 0.25 mmol/L separately. Beas-2b cells were treated with the same concentration and maintained for 30 passages. Treated cells were then analysed in subsequent assays. All experiments were performed with mycoplasma-free cells.

### siRNA transfection

Specific siRNAs (Jima Gene, China) were used for the knockdown of *SREBF1* and *FASN*. A549 and H1975 cells were seeded in 6-well plate and transfected by the 1 µM *SREBF1* or *FASN* siRNA via Lipofectamine 2000. After siRNA transfection for 24 h, the cells were stimulated with 100 µg/mL PM_2.5_ for 48 h. Finally, the expression of *SREBF1* and *FASN* was confirmed by RT-qPCR.

### Cell migration and cell invasion assays

A549 and H1975 cells were seeded into 6-well plates. After treatment, scratch wounds were made by scraping the cell layer in each culture well using the tip of a 200-µL pipette when the cell density reached > 90%. Then, the cells were washed with PBS and cultured for 24 h or more, individually or together, after which three fields were randomly chosen from each scratch wound and visualized by microscopy to evaluate the cell migration ability. The experiments were performed in triplicate.

Invasion assays were performed with Transwell inserts (8-µm pores, Millipore). The upper chambers of the Transwells were precoated with diluted Matrigel (BD Biosciences, Sparks, MD). A total of 10^4^ cells were seeded onto the upper chamber in serum-free medium, and medium containing 10% serum was added to the lower chamber as a chemoattractant. After 24 h of incubation, the upper surface of the insert was wiped with a cotton swab, and cells that migrated to the lower surface were fixed with 4% paraformaldehyde (PFA) and stained with crystal violet. The cells were counted in three random fields per well, and the quantity is expressed as the mean ± SD of triple assessments.

### Colony forming assay

Cells were exposed to the indicated treatments accordingly and seeded into 10 cm dishes (500 cells/dish). After culturing for 14 days, colonies were fixed with 4% PFA and stained with 0.4% crystal violet solution for analysis. Experiments were performed in triplicate.

### RNA-Seq and data analysis

Cells were treated in triplicate per group with PM_2.5_, d-limonene or both. Total RNA was extracted and subsequently sequenced on an Illumina HiSeq 3000 machine. The RNA-Seq reads were aligned to the human genome (GRCh37/hg38) using TopHat version v2.1.0 with the hg38 iGenome annotation guide and default parameters. TopHat was also run permitting novel junctions for the purpose of novel transcript assembly. Cufflinks v2.2.1 was used to assemble transcript models and compare these to the reference hg38 iGenome annotation. Differentially expressed mRNAs were identified in cuffdiff 2.2.1 based on the criterion of being significantly altered in the experimental vs. control condition (P < 0.1 and absolute fold change > 1).

### Electron microscopy

Cells and lung tissues were fixed with 1% osmic acid for 1 h. The samples were transferred into a penetrant prepared according to a ratio of embedding agent and acetone volume of 1:1. After using a 60-nm ultrathin microtome and staining in 1% uranyl acetate lead citrate for double staining, samples were observed and captured with an electron microscope.

### Western blotting

After treatment, the cells and the mouse lung tissues were lysed by RIPA with protease phosphate inhibitor. The proteins were separated by 10% SDS-PAGE and electrotransferred to a Polyvinylidene Difluoride (PVDF) membrane. The PVDF membrane was blocked for 2 h at room temperature with 5% BCA and incubated overnight with rabbit or mouse polyclonal antibodies against GAPDH (CW0100M, CWBIO, China), ACTIN (CW0096, CWBIO, China), ACACA (ab45174, Abcam, UK), FASN (ab22759, Abcam, UK), SCAP (ab190103, Abcam, UK), Collage I (ab138492, Abcam, UK), Vimentin (ab92547, Abcam, UK) and SREBP1 (sc-365513, SCBT-Santa, Japan) at 4 °C. After washing with PBST for 0.5 h, the PVDF membrane was incubated for 2 h with antibody-conjugated goat anti-rabbit IgG or anti-mouse IgG. The PVDF membrane was washed with PBST and then visualized via a protein imaging system.

### Luciferase reporter assay

Luciferase reporter plasmids containing the 3′ untranslated region (3′ UTR) of *SREBP1*—either the wild-type coding sequence region or a sequence with a mutation in the predicted binding site of the *FASN* and *ACACA* genes—were constructed by Jima Gene (China). A549 and H1975 cell lines were seeded in 12-well plates, and then the reporters (1 µg) and *miR-195*-mimics or negative control (NC) (25 nM) were co-transfected into the cells with Lipofectamine 2000. After 24 h, the cells were harvested, and luciferase activities were detected by the Dual Luciferase Reporter Assay Kit.

### RT-qPCR analysis

Total RNA was extracted from cells and tissues after treatment using TRIzol. Subsequently, RNA was reverse-transcribed using PrimeScript™ RT Master Mix. RT-qPCR amplification was performed with an ABI 7500 system with PowerUP™ SYBR™ Green® Master Mix. The expression level of the target gene was normalized to that of GAPDH, and the 2^−ΔΔCT^ method was used to calculate the expression results. Each group included 3 duplicate wells.

### Masson’s trichrome staining

Mouse lung tissues embedded in wax were cut into 6-µm thick slices. The slices were immersed in dimethylbenzene and gradient ethanol for deparaffinization and rehydration. After staining in iron haematoxylin solution, 1% hydrochloric acid alcohol was used for differentiation. The slices were immersed in Ponceau staining solution, phosphomolybdate aqueous solution and aniline blue solution for 10 min, 3 and 6 min, respectively, for redyeing. The slices were sealed with Permount TM mounting medium after dehydration and vitrification in dimethylbenzene and gradient ethanol. Pictures were captured with a microscope, and data were analysed with Image Pro-Plus.

### *miR-195* knockout (KO) mice construction

We used CRISPR/Cas9 technology to construct a *miR-195* KO mouse model via non-homologous recombination. The brief process is as follows: Cas9 mRNA and guide RNA (gRNA) (AAGACTCTACTTTGCTCTGT GGG) were obtained by in vitro transcription; Cas9 mRNA and gRNA were microinjected into the fertilized eggs of C57BL/6J mice to obtain F0 generation mice. After confirmation by PCR product sequencing, the obtained F0 generation mice with target gene deletion were mated with C57BL/6J mice to obtain positive F1 generation mice.

### Animals and treatments

C57BL/6 male mice weighing 22–26 g were purchased from the Experimental Animal Center of Chinese Military Medical Sciences Academy. After 1 week of acclimatization, *miR-195* KO and WT mice were randomly distributed into equal groups, including the control group, PM_2.5_ group and PM_2.5_ plus d-limonene group. The treatment groups were instilled into rear nasal cavities with a 0.1 mL PM_2.5_ suspension, while the control group was instilled with 0.1 mL physiological saline after the mice were anaesthetized with diethyl ether. d-Limonene was administered intragastric at a dose of 600 mg/kg body weight. Exposure was performed once every day, and this practice continued for 2 months. After every treatment, the mice were euthanized, and their lungs were rapidly removed. The mice were sacrificed by cervical dislocation. The removed lungs were placed in liquid nitrogen instantly.

### Sample collection and oil red staining

Human LUAD cancer and corresponding adjacent noncancerous tissues were collected with informed consent from patients who underwent radical resections from Shanghai City and Hebei Province. The tissues were cut into 10-µm-thick slices after flash freezing in liquid nitrogen. The sections were fixed in ice-cold 4% PFA for 30 min and then placed into oil red solution for 10 min at 37 °C after washing in PBS. An 85% propylene glycol solution was used for 2 min for differentiation, and then the sections were rinsed in 2 changes of PBS.

### Recruitment and inclusion/exclusion criteria

Twelve people were recruited into the d-limonene intervention group after strict inclusion and exclusion criteria were met (Trail number: ChiCTR2000030200), and 1 g d-limonene was administered every day for 4 weeks. Ten millilitres of whole blood was captured every 2 weeks, including at baseline. The inclusion criteria were as follows: healthy subjects more than 18 years old; body mass index (BMI) more than 18.5 kg/m^3^; no history of allergy to citrus fruits; and voluntarily participation with sign informed consent. The exclusion criteria were as follows: the presence of acute or chronic gastrointestinal diseases; intake of more than 50 g citrus fruits 1 day in a recent week; the presence of heart, liver, kidney or haematopoietic system disease or other serious diseases; severe allergic constitution; pregnant or lactating women; the use of anti-inflammatory drugs; surgery was not scheduled in the next 3 months.

### Cohort analysis

We constructed a PM_2.5_ exposure cohort based on the SuValue database. The SuValue database included 221 hospitals from 23 provinces, municipalities or autonomous regions across China. The 221 hospitals included more than one million patients from 176 general hospitals, 28 traditional Chinese medicine hospitals, 14 maternal and childcare hospitals and 3 specialized hospitals. People in this cohort had at least two tests for triglycerides (TGs), which are the main component of lipid droplets. The PM_2.5_ dataset of each province across China was collected from the Atmospheric Composition Analysis Group.

### Statistical analysis

The results of laboratory experiments are presented as the mean ± SD. Student’s t test was used to compare means between two groups, and ANOVA was employed for comparison of more than two groups when samples were normally distributed. Otherwise, the rank sum test was used for comparison between the two groups, and the Kruskal–Wallis test was used for comparison between multiple groups. All statistical analyses were performed using Statistical Package for the Social Sciences (SPSS) software (version 19.0) and GraphPad Prism version 8.0 (GraphPad Software, San Diego CA, USA). P < 0.05 indicates a significant difference (* means P < 0.05, ** means P < 0.01, *** means P < 0.001). All experiments were repeated three times.

## Results

### PM_2.5_ chemical characteristics

As shown in Table 1, the dominant components of metal ions and part non-metallic ions in PM_2.5_ were Al (1255.33 ± 5.51 ng/mg), Ca (9351.68 ± 69.97 ng/mg), Fe (2691.15 ± 20.84 ng/mg), Na (1567.68 ± 13.40 ng/mg), K (1567.43 ± 2.98 ng/mg), Mg (1074.41 ± 6.25 ng/mg) and S (12688.06 ± 66.99 ng/mg). The average concentrations of OC and EC in the PM_2.5_ were 527.11 ± 144.62 µg/mg and 119.71 ± 50.11 µg/mg, and the OC/EC ratio was about 4.61 ± 1.16. Among the total of 16 PAHs elements measured, Naphthalene, Acenaphthylene and Pyrene were the most abundant elements in the PM_2.5_.

**Table 1:** Metals, Carbon and organic compositions detected in PM_2.5_

Metals	Concentration (ng/mg)	OC\EC	Concentration (µg/mg)
Al	1255.33 ± 5.51	OC	527.11 ± 144.62
As	11.53 ± 0.46	EC	119.71 ± 50.11
B	28.59 ± 0.39	OC/EC	4.61 ± 1.16
Ba	95.20 ± 1.00	**PAHs**	**Concentration** (ng/mg)
Be	0.11 ± 0.00		
Bi	1.69 ± 0.97	Naphthalene	32.38 ± 1.34
Ca	9351.68 ± 69.97	Acenaphthylene	1.16 ± 0.49
Cd	2.96 ± 0.01	Acenaphthene	0.56 ± 0.32
Co	2.02 ± 0.15	Fluorene	0.42 ± 0.30
Cu	94.05 ± 1.18	Phenanthrene	0.14 ± 0.06
Fe	2691.15 ± 20.84	Anthracene	0.01 ± 0.01
Hg	1.16 ± 0.07	Fluoranthene	0.21 ± 0.15
K	1567.43 ± 2.98	Pyrene	0.66 ± 0.14
Li	4.28 ± 0.21	Benzo [A] anthracene	0.07 ± 0.01
Mg	1074.41 ± 6.25	Chrysene	0.16 ± 0.12
Mn	181.71 ± 1.58	Benzo [B] fluoranthene	0.39 ± 0.16
Mo	8.43 ± 0.04	Benzo [K] fluoranthene	0.15 ± 0.11
Na	1567.68 ± 13.40	Benzo [A] pyrene	0.15 ± 0.06
Ni	27.37 ± 0.16	Indene (1, 2, 3-cd) pyene	0.39 ± 0.06
P	291.58 ± 0.15	Diphenyl anthracene (A, H)	0.06 ± 0.08
Pb	106.62 ± 0.70	Benzo [G, H, I] pyrene	0.17 ± 0.13
S	12688.06 ± 66.99		
Sb	14.08 ± 0.58		
Se	12.61 ± 1.52		
Si	1019.26 ± 7.59		
Sn	13.56 ± 0.49		
Sr	35.02 ± 0.33		
Ti	62.17 ± 0.22		
V	73.25 ± 0.36		
W	18.56 ± 1.70		
Zn	493.88 ± 0.89		
Zr	4.23 ± 0.39		

*OC* organic carbon, *EC* elemental carbon, *PAHs* Polycyclic Aromatic Hydrocarbons

### PM_2.5_ promotes the aggressive phenotypes of lung cancer cells and pulmonary fibrosis of mice

**Fig. 1:**
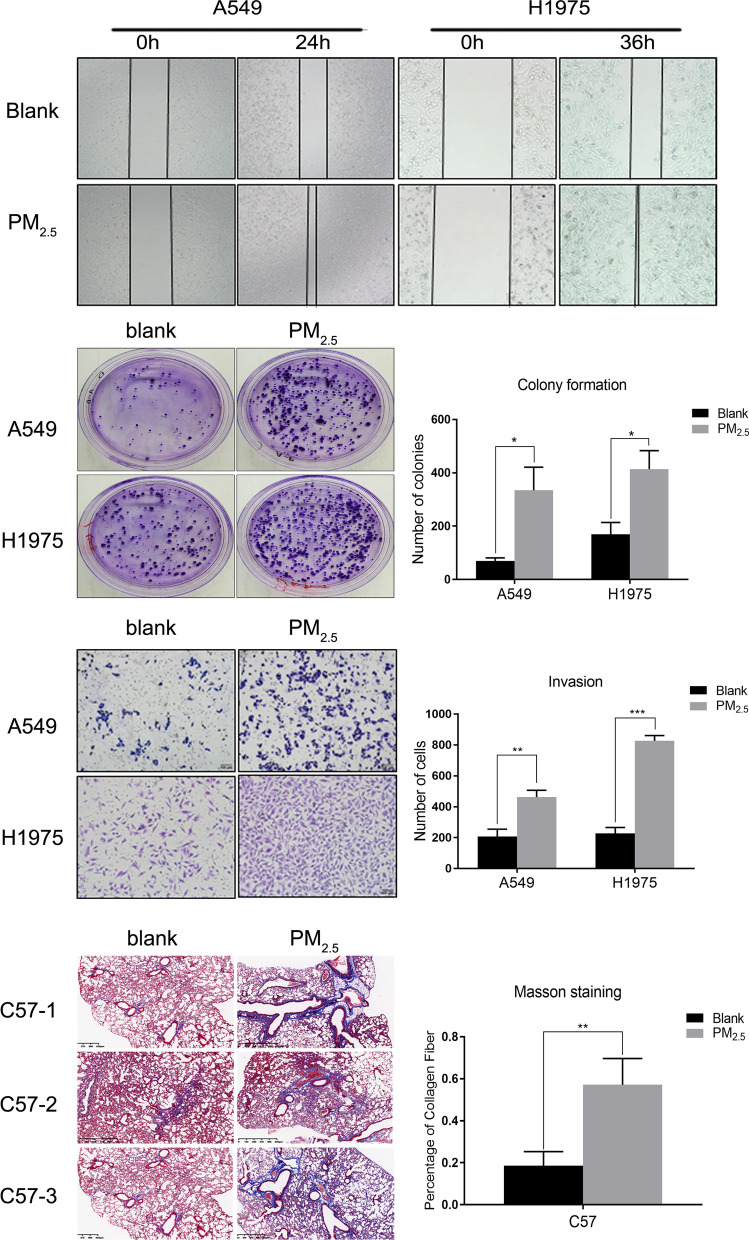
LUAD cells became more aggressive and WT mice got pulmonary fibrosis after PM_2.5_ exposure. **A**–**C** PM_2.5_ treatment increased the migration, colony formation and invasion of A549 and H1975 cells according to the wound healing assays, plate colony formation and Transwell experiments. **D** Masson’s trichrome staining showed that pulmonary fibrosis caused by PM_2.5_ exposure in C57BL/6 mice (N = 6). *P < 0.05; **P < 0.01; ***P < 0.001

To study the toxic effect of PM_2.5_ on lung cancer, we treated A549 and H1975 LUAD cells with PM_2.5_. After treatment, cell migration assays showed that A549 and H1975 treated with PM_2.5_ migrated faster than those untreated cells, although A549 showed large variation in migration at 24 h (Fig. 1A). Colony formation assays showed increased colony formation in both A549 and H1975 after the cells were treated with PM_2.5_ for 14 days (Fig. 1B). Transwell assays demonstrated that A549 and H1975 cells treated with PM_2.5_ became more aggressive, and the number of cells penetrating through the Matrigel membrane increased by 2–4 times (Fig. 1C). Meanwhile, Masson’s trichrome staining showed that pulmonary fibrosis occurred in C57BL/6 mice obviously after long-term exposure to PM_2.5_ (Fig. 1D).

### PM_2.5_ accumulates lipid droplets in LUAD cells after PM_2.5_ exposure

**Fig. 2:**
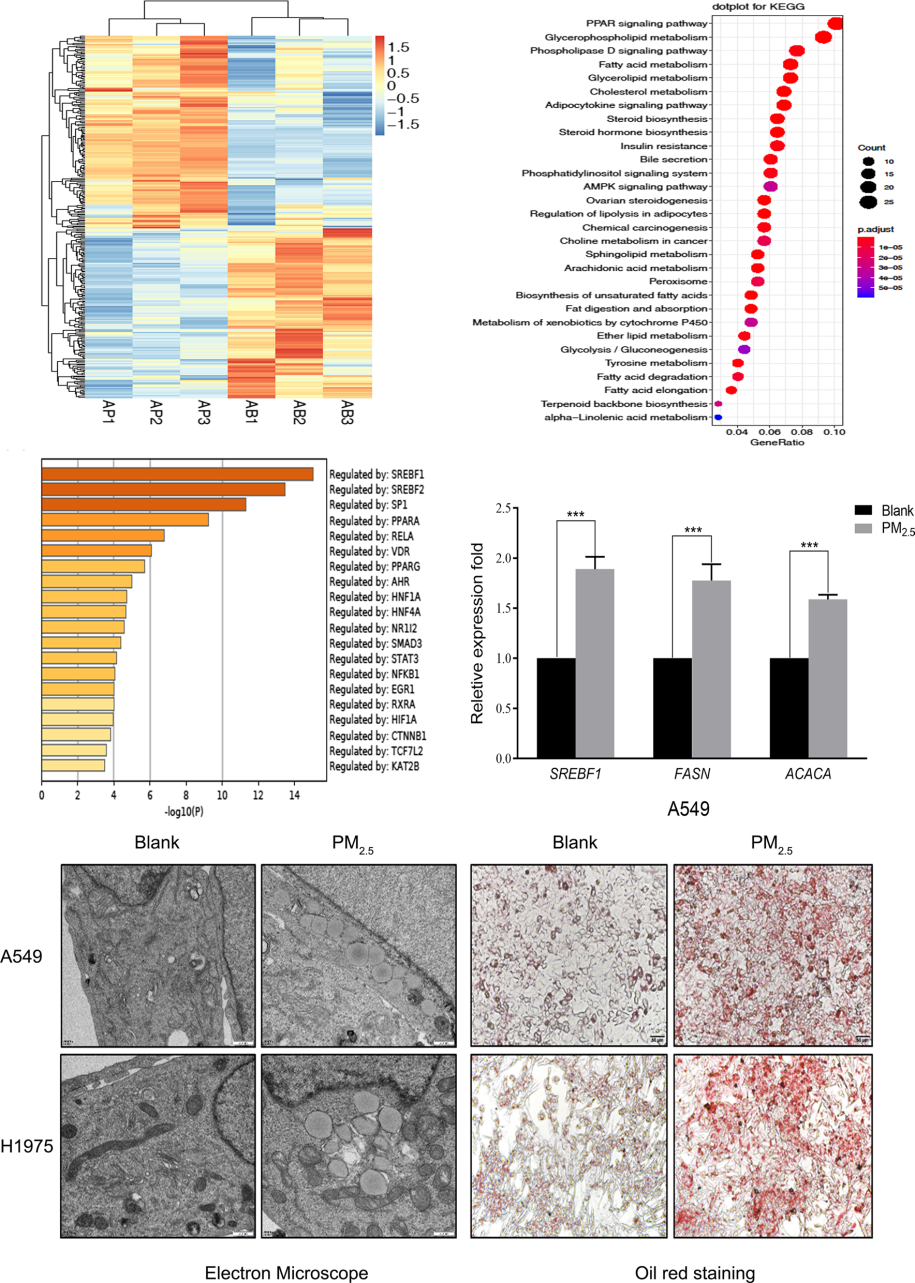
Transcriptomic analysis showed that lipogenic genes were upregulated and lipid droplets were clearly detected after PM_2.5_ treatment of A549 and H1975 cells. **A** Heat map showing the differentially expressed genes between A549 cells treated with and without PM_2.5_ exposure. **B** KEGG results showing enrichment of the DEGs in the lipid metabolism pathway. **C** Results of transcriptional regulatory relationships according to sentence-based text mining. **D** Verification of the target genes by RT-qPCR. *SBEBF1*, *FASN* and *ACACA* were upregulated after PM_2.5_ exposure. **E** Compared to the untreated cells (blank), lipid droplets were substantially increased in A549 and H1975 after the cells were treated with PM_2.5_, as shown by electron microscope (Scale bar = 500 nm) and oil red O staining (Scale bar = 50 μm). *AP* A549-blank group, *AB* A549-PM_2.5_ exposure group. ***P < 0.001

To understand the mechanisms by which PM_2.5_ promotes the aggressive phenotypes of lung cancer cells and pulmonary fibrosis of mice, we analyzed the transcriptomes of A549 cells with RNA-seq after PM_2.5_ treatment. The transcriptomic analysis revealed 263 differentially expressed genes (DEGs) between cells treated with and without PM_2.5_. Of these DEGs, 148 were upregulated, and 115 were downregulated (Fig. 2A). Using the Kyoto Encyclopedia of Genes and Genomes (KEGG) algorithm to interrogate the DEG profiles, we found significant signal enrichment in fatty acid metabolism (Fig. 2B). The top two genes involved were SREBFs (Fig. 2C). We confirmed these findings with RT-qPCR analysis on *SREBF1* and its downstream targets, *FASN* and *ACACA*, all of which were upregulated significantly after PM_2.5_ treatment (Fig. 2D). Furthermore, we observed clear increases in lipid droplets in both cell lines evaluated with electron microscope or Oil Red O staining compared to those untreated cells after the treatment (Fig. 2E).

### PM_2.5_ promotes N-SREBP1 translocation into the nucleus, inducing lipid droplet formation and LUAD progression

**Fig. 3:**
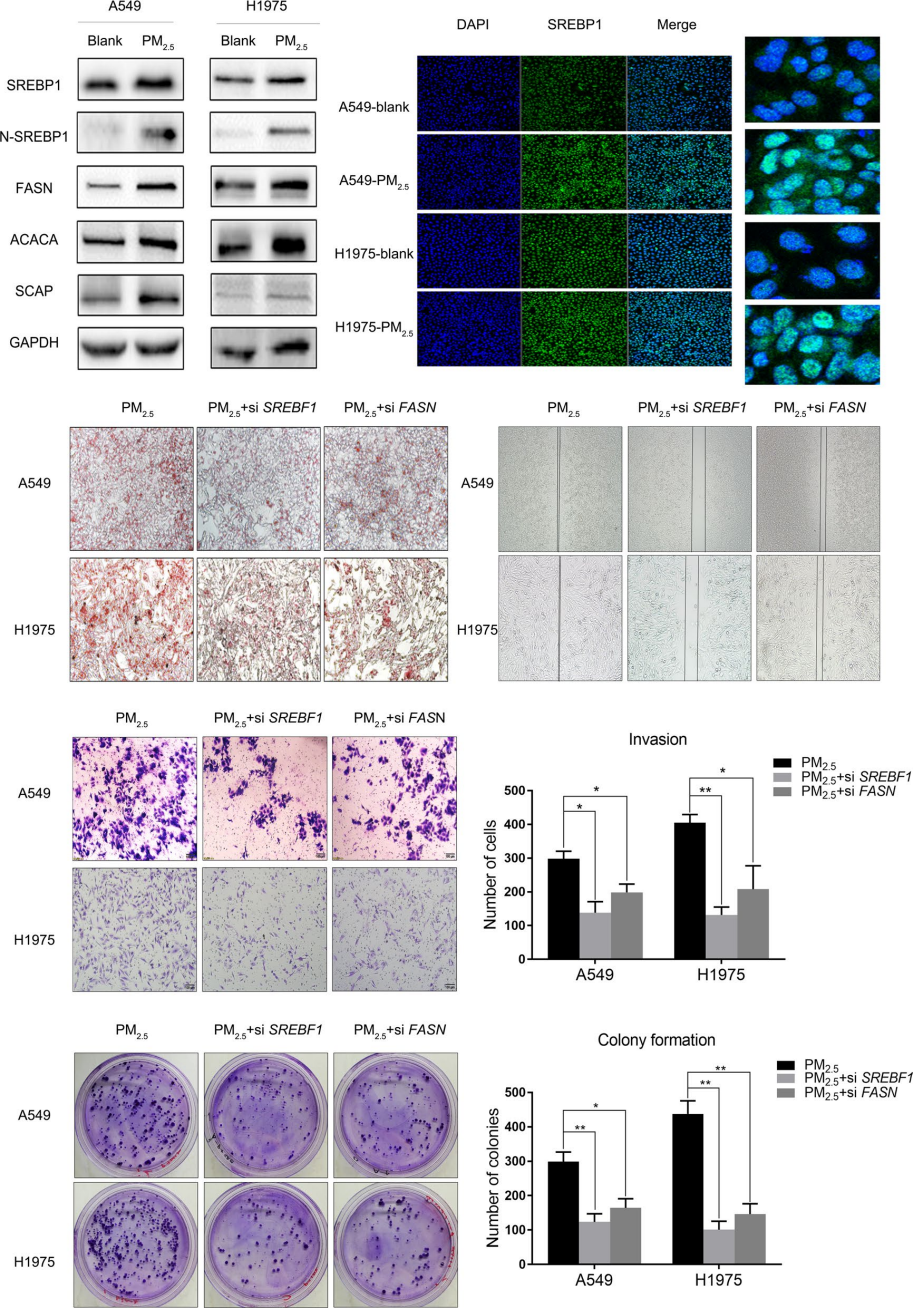
PM_2.5_ promoted N-SREBP1 translocation into the nucleus, inducing lipid droplet formation. **A** Western blot analysis showed that N-SREBP1, FASN, ACACA and SCAP proteins were upregulated in H1975 and A549 cells after PM_2.5_ treatment. **B** Fluorescent staining showed that N-SREBP1 in the nucleus of A549 and H1975 cells was increased significantly after PM_2.5 _treatment. **C** Oil red O staining showed that the production of intracellular lipid droplets was reduced after *SREBF1* or *FASN* was knocked down. **D**–**F** After *SREBF1* or *FASN* was knocked down, the migration, colony formation and invasion abilities induced by PM_2.5_ treatment were inhibited. Scale bar = 50 μm. *P < 0.05; **P < 0.01

Following the above findings, we speculate that PM_2.5_ may upregulate the expression of SREBP1 and its downstream genes, which leads to changes in lipid metabolism, resulting in increases in lipid droplets and aggressive cell behaviors. However, the level of total SREBP1 protein was not elevated obviously after PM_2.5_ exposure in either lung cancer cell lines based on western blot analysis, while its downstream proteins FASN and ACACA were clearly upregulated (Fig. 3a). Using fluorescence immunohistochemical staining for SREBP1, we found that the protein was enriched in the cell nucleus (Fig. 3B). Western blot analysis showed that N-SREBP1 and SREBP cleavage activating protein (SCAP) were upregulated after PM_2.5_ exposure (Fig. 3A). Next, we used siRNA to knock down *SREBF1* and the main downstream gene *FASN*. Oil Red O staining showed that the number of intracellular lipid droplets in the knockdown cells after PM_2.5_ exposure was reduced (Fig. 3C). Additionally, knocking down the two genes inhibited the PM_2.5_-induced increases in cell invasion, migration and colony formation (Fig. 3D–F), suggesting that SREBP1 plays an important role in the accumulation of lipid droplets and the aggression of lung cancer cells induced by PM_2.5_ exposure.

### d-Limonene inhibits PM_2.5_-induced lipid droplets accumulation in vivo and in vitro

**Fig. 4:**
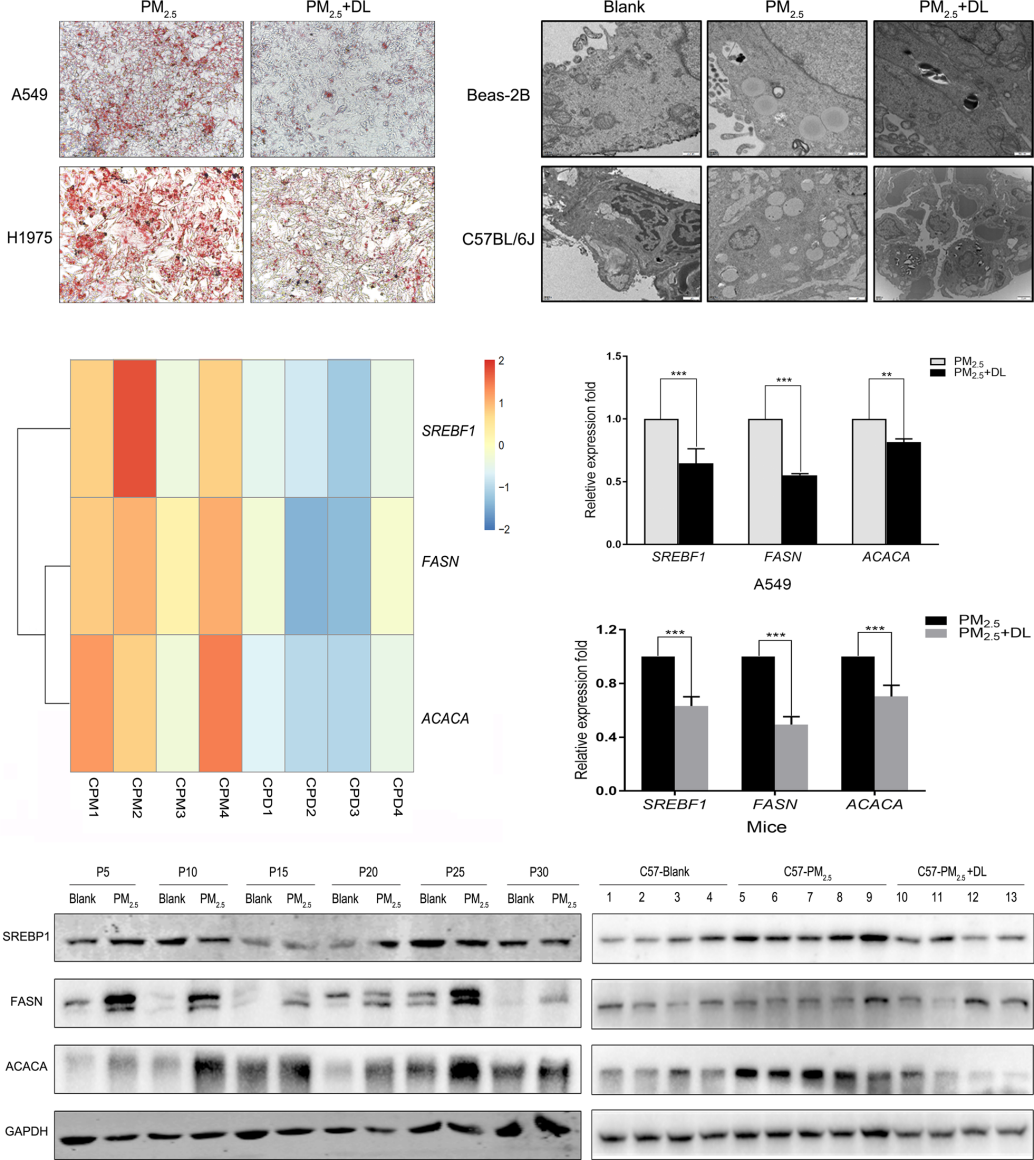
d-Limonene inhibited lipid droplets accumulation caused by PM_2.5_ exposure in vivo and in vitro. **A** Oil red O staining showed that PM_2.5_ exposure-induced lipid droplet formation was reduced after d-limonene treatment in LUAD cells. **B** Electron microscopic views of lipid droplets in normal lung epithelial cells (Beas-2b) and normal mouse lung tissue after long-term exposure to PM_2.5_. The number of lipid droplets decreased after treatment with d-limonene. **C** RNA-Seq analysis showed that PM_2.5_-induced upregulation of *Srebf1*, *Fasn* and *Acaca* was suppressed after treatment with d-limonene in mice. **D**, **E** PM_2.5_-induced upregulation of *SREBF1*, *ACACA* and *FASN* was inhibited by d-limonene intervention in A549 cell and C57BL/6 mice according to RT-qPCR. **F** Western blot analysis showed that PM_2.5_-induced upregulation of SREBF1, ACACA, and FASN was attenuated by treatment with D-limonene in Beas-2b cells and normal mouse lung tissue (N = 4, 5, 4). Scale bar = 500 nm. **P < 0.01; ***P < 0.001. CPM: C57BL/6 − PM_2.5_ exposure group; CPD: C57BL/6 − PM_2.5_ exposure + d-limonene group

To investigate whether d-limonene played a protective role in lipid metabolism disorder caused by PM_2.5_ exposure, cells and mice were treated with d-limonene when exposed to PM_2.5_ simultaneously. After treatment with d-limonene, lipid droplet formation induced by PM_2.5_ exposure was decreased dramatically in A549 and H1975 cells (Fig. 4A). Beas-2b cells were cultured under PM_2.5_ exposure for 30 passages. The PM_2.5_ treatment led to significant lipid droplets accumulation in the cells, and this phenomenon did not appear after the cells were treated with both PM_2.5_ and d-limonene (Fig. 4B). We also found a large amount of lipid droplets in the lung tissue of C57BL/6 mice after the animals were treated with PM_2.5_ for 60 days, but no lipid droplets were observed in the animals when d-limonene was orally administered simultaneously with PM_2.5_ inhalation (Fig. 4B). RNA-Seq analysis was performed on the lung tissues of mice treated with PM_2.5_ or PM_2.5_ plus d-limonene. The expression of *Srebf1*, *Fasn* and *Acaca* upregulated by PM_2.5_ exposure were suppressed by d-limonene treatment (Fig. 4C). Similar results were also seen in LUAD cells, and the findings were verified by RT-qPCR (Fig. 4D, E).

To assess time-dependent variations, we collected Beas-2b cell samples every 5 passages, and the samples were analysed with western blotting. As shown in Fig. 4f, the expression of SREBP1, FASN and ACACA were significantly higher in PM_2.5_-exposed than in control groups, and the increases appeared as early as in the fifth generation of cells. These proteins were also upregulated in the mouse lung tissue after a long period of exposure to PM_2.5_. Similar to the observations in LUAD cells, the expression of SREBP1, FASN and ACACA, which were promoted by PM_2.5_ exposure, were inhibited by d-limonene treatment to the mice (Fig. 4F).

### d-Limonene inhibits PM_2.5_-induced the occurrence and progression of LUAD by upregulating *miR-195*

**Fig. 5:**
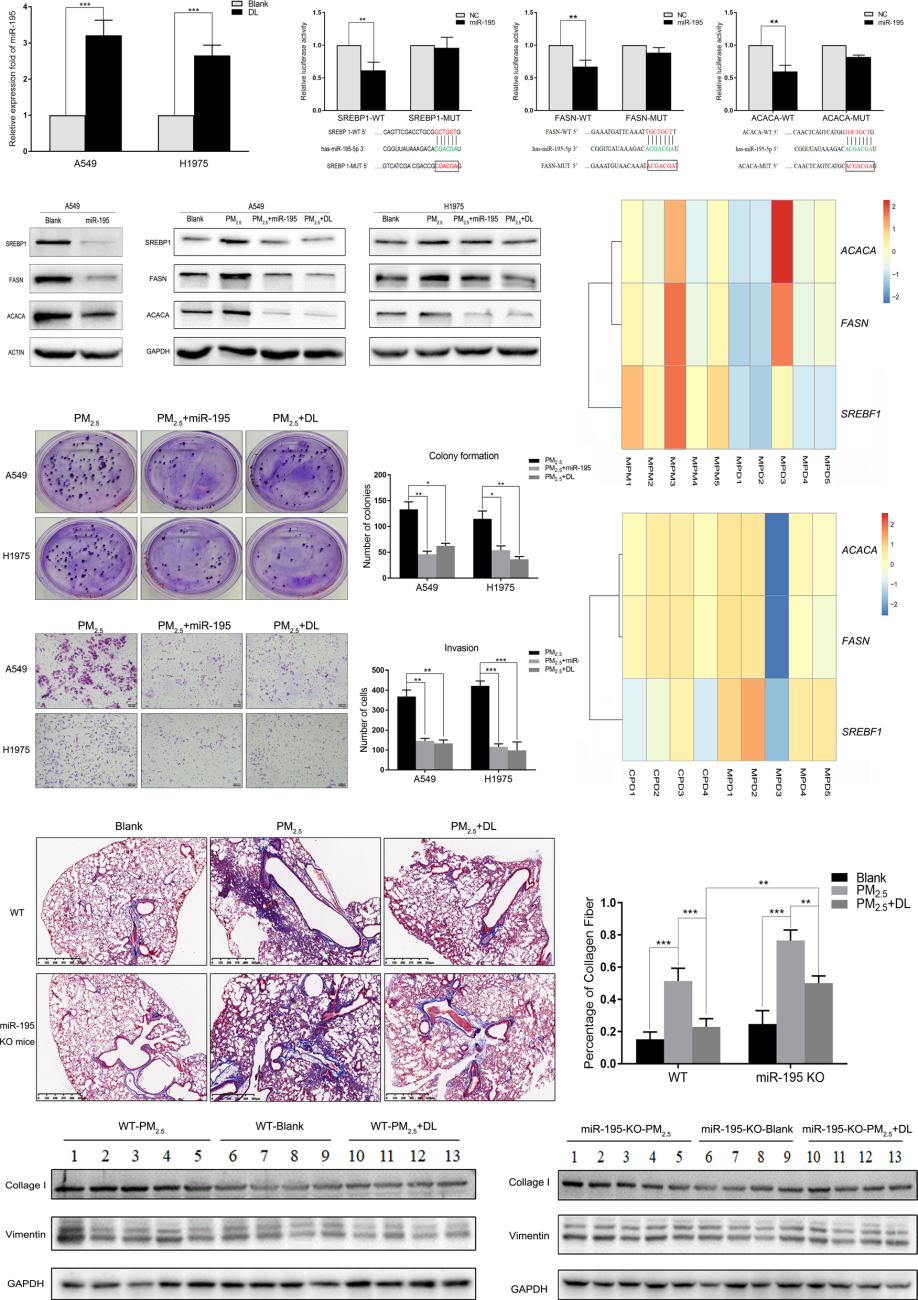
d-Limonene inhibited lung cancer progression and pulmonary fibrosis by attenuating lipid metabolism disorder caused by PM_2.5_ exposure. **A** The expression of *miR-195* increased after treatment with d-limonene. **B** Dual luciferase experiments showed the binding of *miR-195* with *SREBF1, FASN* and *ACACA* mRNAs. **C** The protein expression of SREBP1, FASN and ACACA was inhibited by *miR-195*. **D** Western blot analysis showed that SREBP1, FASN and ACACA upregulated by PM_2.5_ exposure were attenuated by d-limonene or *miR-195* treatment. **E**, **F** Plate colony formation and Transwell experiments showed that PM_2.5_-induced proliferation and invasion of lung cancer cells were inhibited by d-limonene or *miR-195* treatment. **G**, **H** The upregulation of *SREBF1, ACACA* and *FASN* by PM_2.5_ in *miR-195* KO mice was attenuated after d-limonene treatment (**G**), and the attenuation was more evident in WT mice (**H**). **I** Masson’s trichrome staining showed that d-limonene repaired the fibrosis caused by PM_2.5_ exposure, and the level of pulmonary fibrosis in *miR-195* KO mice treated with d-limonene was significantly higher than that of WT mice (N = 6). **J**, **K** Western blot analysis showed that PM_2.5_-induced upregulation of collagen I and vimentin was inhibited by d-limonene in WT and *miR-195* KO mice (N = 4, 5, 4, separately). *P < 0.05; **P < 0.01; ***P < 0.001. MPM: *miR-195* KO mice − PM_2.5_ exposure group. MPD: *miR-195* KO mice − PM_2.5_ exposure + d-limonene group. CPD: C57BL/6J mice − PM_2.5_ exposure + d-limonene group

To investigate the protective mechanisms of d-limonene played in lung cancer, we analysed A549 and H1975 cells treated with or without d-limonene. Interestingly, we found that the expression of *miR-195*, a tumor suppressor, was substantially upregulated after d-limonene treatment (Fig. 5A). Using miRWalk [21] and miRSystem [22], we found that *miR-195* was predicted to interact with *SREBF1* in the CDS region when energy < − 20, accessibility < 0.0001, me < − 8. The in silico prediction also indicated that the miRNA could interact with *FASN* and *ACACA* mRNAs at their 3′ UTRs. The prediction that the seed sequence of *miR-195* can directly bind to the CDS region of *SREBF1* and the 3′ UTR of *FASN* and *ACACA* was verified by our dual luciferase assays. Our experiments showed that the relative luciferase activity was reduced in the WT groups compared with the mutant groups (Fig. 5B). After *miR-195* was overexpressed in A549 cells, western blot analysis showed that SREBP1, FASN and ACACA were significantly inhibited (Fig. 5C).

To verify the effects of d-limonene and *miR-195* on inhibiting lipid metabolism disorder caused by PM_2.5_ exposure, A549 and H1975 cells were treated with or without *miR-195* or d-limonene after PM_2.5_ exposure. Western blot analysis showed that d-limonene or *miR-195* decreased the protein levels of SREBP1, FASN and ACACA, which were upregulated in A549 and H1975 cells after PM_2.5_ exposure (Fig. 5D). Colony formation and Transwell experiments showed that both d-limonene and *miR-195* inhibited the PM_2.5_ exposure-induced proliferation and invasion of lung cancer cells (Fig. 5E, F). The RNA-Seq results of *miR-195* KO treated with PM_2.5_ with or without d-limonene showed that d-limonene inhibited the increases in expression of *Srebf1*, *Fasn* and *Acaca* induced by PM_2.5_ exposure in the KO mice (Fig. 5G), but the inhibitory effect was weaker than that in the WT mice (Fig. 5H). Furthermore, Masson’s trichrome staining and western blotting analysis revealed that pulmonary fibrosis occurred in both WT mice and *miR-195* KO mice after long-term exposure to PM_2.5_. This phenotype could be rescued by d-limonene treatment. The level of pulmonary fibrosis in *miR-195* KO mice treated with d-limonene was significantly higher than that in WT mice (Fig. 5I–K). The above results suggest that the presence of *miR-195* in WT mice facilitates the d-limonene’s suppression and attenuation on PM_2.5_-induced lipid-related gene expression and of pulmonary fibrosis development, respectively.

### PM_2.5_ exposure increased TG levels in human plasma and lipid droplets in the lung tissue of cancer patients

**Fig. 6:**
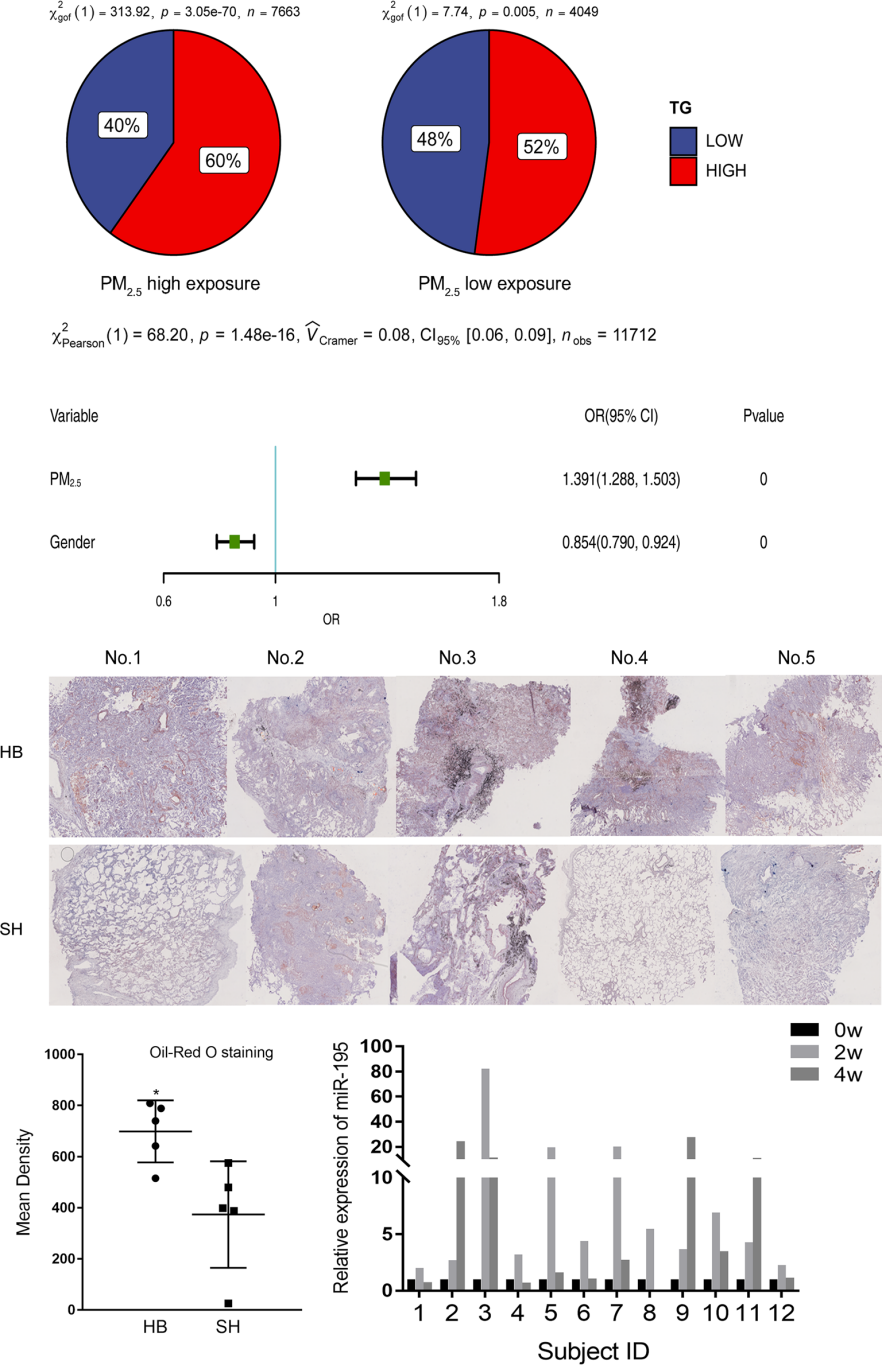
Serum levels of TG and tissue levels of lipid droplets in people living in regions with different PM_2.5_ pollution.** A** 60% of people living in regions with high PM_2.5_ pollution had higher serum levels of TG, whereas 52% of people living in low pollution areas had the same levels of TG. **B** Multivariate logistic regression analysis showed that PM_2.5_ exposure were associated with high TG levels. **C**, **D** Oil red O staining results showed more lipid droplets in the lung tissue of cancer patients living in high than in low PM_2.5_ pollution areas (N = 5). *P < 0.05. **E** Changes in *miR-195* levels in people taking d-limonene orally (N = 12). *SH* Shanghai City, *HB* Hebei Province

We assembled a cohort of 11,712 lung cancer patients with information on serum TG levels and their residences where air monitoring data were available on ambient PM_2.5_. Based on their residence, 7663 individuals were classified as living in high exposure areas, and 4049 were in low exposure regions. More patients living in high exposure regions had higher TG levels than those living low exposure regions, 60% vs. 52% (Fig. 6A). Multivariate logistic regression analysis showed that high PM_2.5_ exposure were associated with elevated serum TG levels after adjusting for sex and age (HR = 1.39, 95% CI 1.29–1.50) (Fig. 6B). Compared to those in Shanghai City, lung cancer patients from Hebei Province where PM_2.5_ pollution was higher than Shanghai, had more lipid droplets in their lung tissues according to the Oil Red O staining (Fig. 6C, D). A single-arm d-limonene intervention trial was conducted in twelve people. Over a 4-week oral administration of d-limonene, individuals in the trial had substantial increases in serum levels of *miR-195* (Fig. 6E).

## Discussion

PM_2.5_ exposure is an important environmental risk factor for pulmonary injury and lung cancer mortality [23–26], but the mechanism is unclear. The lack of understanding impedes our effort on prevention. In our previous studies, we found that PM_2.5_ influenced lung cancer cells by increasing oxidative stress and autophagy and dysregulating certain long non-coding RNAs (lncRNAs) [19, 20]. Other researchers reported that PM_2.5_ upregulated Wnt3a/β‑catenin pathway and IL-17a signal pathway or reduced the levels of 15-lipoxygenases, which influenced the progression of lung cancer [27–29]. In this study, we found a notable phenomenon that long-term environmental exposure to ambient PM_2.5_ leads to enlarged lipid droplet accumulation in pulmonary cells and then identified a possible mechanism that may explain the phenomenon. We found that PM_2.5_-induced lipid droplet accumulation was caused by the translocation of SREBP1 from cytoplasm to nucleus where it upregulated the expression of FASN and ACACA and activated de novo lipogenesis pathway resulting in dysregulation of lipid metabolism.

Lipid droplets are metabolically active organelles with a neutral lipid core (predominantly triglycerides and cholesterol esters) surrounded by a monolayer of phospholipids and proteins [30]. More recently, they are well known as critical organelles involved in numerous biological functions, especially in the field of liver-related diseases [31]. Lipid droplets in more than 5% of the hepatocytes is a significant characteristic of Non-alcoholic fatty liver disease (NAFLD), which can be followed by the development of hepatic fibrosis, liver cirrhosis and even hepatocellular carcinoma [32, 33]. Lipid droplet have been reported to be involved in several types of cancer [34, 35], including pancreatic ductal adenocarcinoma [36], colorectal cancer [37], breast cancer [38], and ovarian cancer [39]. Zhou and Huang et al. found that lipid droplets accumulated in lung cancer cells could induce EGFR-TKI resistance, which would be overcame by co-administration of lipid metabolism inhibitors and EGFR-TKIs [40, 41].

We found that lipid droplets became increasingly larger in lung cancer cells after exposure to PM_2.5_ (Fig. 2E). Lipid droplets also appeared in normal lung epithelial cells and mouse lung tissues after PM_2.5_ exposure (Fig. 4B). They were all accompanied by increased malignant cellular behaviors or fibrosis (Fig. 5I). Our patient cohort also showed that circulating levels of TG were higher in people living in PM_2.5_ heavily polluted regions than in those living in low polluted areas (Fig. 6A). It is similar to the data from cohort studies in Chinese rural population and Korean soldiers, PM_2.5_ exposure are associated with higher blood TG levels [42–44]. Analysis of tumor specimens from lung cancer patients living in Hebei Province, where there is heavy ambient PM_2.5_ pollution due to the presence of many iron and steel mills, showed more lipid droplets in their tissue samples compared to those living in Shanghai, confirming that PM_2.5_ exposure may disrupt lipid metabolism and induce lipid droplet accumulation in human lung tissues.

Lipid droplet accumulation induced by de novo lipogenesis is an important component of lipid metabolism reprogramming, which is widely involved in the development of cancers [45, 46]. SREBP-1 is a key molecule in regulating the production of fatty acids and triglycerides through stimulating the expression of FASN and ACACA, which increases cancer cell viability and promotes tumor growth [47]. Inhibition of de novo fatty acid synthesis suppresses tumor growth of non-small-cell lung cancer in preclinical model [48]. In comparison of cell transcriptomes with and without PM_2.5_ treatment, we found that de novo lipogenesis-related genes, including *SBEBF1*, *FASN* and *ACACA*, were differentially expressed as part of the top signature upregulated by PM_2.5_ (Fig. 2). Our follow-up experiments showed that PM_2.5_ exposure led to the nuclear entry of N-SREBP1 which upregulated FASN and ACACA. Interestingly, the protein expression of SREBP1 increased in normal cells when exposed to PM_2.5_ from P5 to P30 (Fig. 4F), which was a little higher than that occurred in cancer cells (Fig. 3A). The deterioration of cancer cells urgently needs more energy and material support. Therefore, when exposed to PM_2.5_, more SREBP1 was cleaved and transported into the nucleus rapidly to promote the generation of triglycerides and lipid droplets, which promoted the invasion, migration and proliferation of cancer cells. Since the resistance of normal cells and deterioration required an accumulation process, SREBF1 upregulated after PM_2.5_ exposure, and the protein expression level of SREBP1 was even a little higher than that of cancer cells. In vitro experiments demonstrated that lipid droplets were decreased, and cell invasion, migration and proliferation were inhibited when we used siRNA to knock down the expression of *SREBF1* or *FASN*. These observations suggest that dysregulated lipid metabolism induced lipid droplets accumulation after PM_2.5_ exposure may play an important role in the occurrence and progression of lung cancer and that blocking or correcting lipid metabolism dysregulation may be a potential intervention strategy for reducing lung cancer risk associated with PM_2.5_ exposure.

d-Limonene has been found to regulate lipid metabolism in hepatic cells [49]. Morse et al. found that d-limonene could inhibit the metabolic activation of nitrosamine and thus prevent the development of NNK-induced lung cancer through its interaction with cytochrome P450 detoxification enzymes [50]. We reported previously that d-limonene exerts its antitumor activity by inducing autophagy and apoptosis and by regulating lipid metabolism in cancer cells [51–53]. In this study, we further demonstrated that d-limonene inhibited the expression of *SBEBF1*, *FASN* and *ACACA* and decreased lipid droplet accumulation in LUAD cells. d-Limonene could also suppress the colony formation, proliferation, invasion, and migration of lung cancer cells. These results suggest that d-limonene may target the genes affected by PM_2.5_ and regulate the synthesis and metabolism of lipid droplets.

Furthermore, we found that the expression of *miR-195* was increased significantly when the A549 and H1975 cell lines were treated with d-limonene. This miRNA reported by us and others before can suppress the growth of lung cancer and other cancers, including acute lymphoblastic leukaemia, triple-negative breast, liver, glioblastoma, prostate, stomach and colorectal cancers [54–59]. We also found that *miR-195* could directly bind to the mRNAs of *SREBF1*, *FASN* and *ACACA* in lung cancer cells resulting in decreases in lipid droplet formation induced by PM_2.5_ exposure. In addition to inhibiting cancer cell growth, migration and invasion induced by PM_2.5_ exposure, d-limonene may also prevent or slower the development of lung cancer. Our study showed that d-limonene treatment could block or slow the formation of pulmonary fibrosis caused by long-term PM_2.5_ exposure both in vivo and in vitro.

To understand the significance of *miR-195* in d-limonene intervention, a *miR-195* KO mouse model was established. As shown in Fig. 5, when *miR-195* was knocked out, the effect of d-limonene became weaker, and more fibrosis was indicated by high collagen fibre staining and collagen I expression in the animals compared to the wide type mice. Li et al. found that *miR-195* may serve as a biomarker to assist the diagnosis of lung cancer and classification of high-risk populations [60]. In our small trial, we found that the expression of *miR-195* was dramatically increased in human serum after d-limonene treatment, which suggests that *miR-195* may serve as a potential biomarker for monitoring the effect of d-limonene administration.

## Conclusion

In summary, our study showed that PM_2.5_ exposure caused accumulation of lipid droplets in lung cancer cells, normal lung epithelial cells and normal pulmonary tissue, which promoted pulmonary fibrosis and progression of lung adenocarcinoma. This phenomenon resulted from PM_2.5_-induced dysregulation of several proteins (SREBP1, FASN and ACACA) which control de novo lipogenesis. Our study also showed that a monoterpenoid D-limonene could block or reduce the disruption of lipid metabolism caused by PM_2.5_ exposure, indicating its potential value as a chemopreventive agent for reducing the risk of lung cancer associated with PM_2.5_ exposure. Finally, we found that *miR-195* could mediate the effect of d-limonene on lipid metabolism, suggesting that the miRNA may serve as a biomarker to monitor the use of d-limonene.

## Data Availability

The datasets generated/analysed in the present study are available upon reasonable request from the corresponding author.

## Abbreviations

3′ UTR: 3′ Untranslated region
ACACA: Acetyl-CoA carboxylase
BMI: Body mass index
COPD: Chronic obstructive pulmonary disease
DEGs: Differentially expressed genes
FASN: Fatty acid synthase
gRNA: Guide RNA
KEGG: Kyoto Encyclopedia of Genes and Genomes
KO: Knock out
LUAD: Lung adenocarcinoma
LUSC: Lung squamous cell carcinoma
NASH: Non-alcoholic steatohepatitis
PM_2.5_: Fine particulate matter
SREBP1: Sterol regulatory element-binding protein-1
TG: Triglyceride
